# Prevention and Response Strategies for Sexual Violence in Indonesian State Universities: A Multi-Case Study

**DOI:** 10.5964/sotrap.15201

**Published:** 2026-08-06

**Authors:** St. Rodliyah, St. Sariroh

**Affiliations:** 1 *Management of Islamic Education, State Islamic University of Kiai Haji Achmad Siddiq, Jember, Indonesia*; 2 *Constitutional Law Study Program, State Islamic University of Kiai Haji Achmad Siddiq, Jember, Indonesia*; University Medical Center Mainz, Mainz, Germany; University of Kassel, Kassel, Germany

**Keywords:** strategy, sexual violence, Indonesian state universities, Indonesia

## Abstract

Sexual violence in higher education has become a persistent institutional and legal problem in Indonesia, according to the Women's Commission, there were 45,069 reported cases in 2020, yet prevention and response mechanisms vary considerably across universities. This study examines the strategies used by Indonesian state universities to prevent and respond to sexual violence against women and identifies institutional gaps that limit their effectiveness. Using a descriptive qualitative case-study design, the research was conducted in state universities under the Ministry of Religious Affairs and the Ministry of Education, Culture, Research, and Technology. Data were collected through observation, semi-structured interviews, and document analysis involving heads of centres for gender and child studies, PPKS task-force members, legal-aid officers, lecturers, students, and campus units responsible for sexual-violence prevention and response. The findings show that universities have developed multiple initiatives, including institutional regulations, standard operating procedures, PPKS task forces, gender and child study centres, complaint and legal-aid services, awareness programmes, peer-support groups, faculty-level focal points, and gender-responsive learning activities. Case-responding mechanisms generally include reporting, protection, assistance, sanctioning, and victim recovery through medical, psychological, legal, spiritual, and academic support. However, implementation remains uneven. Major weaknesses include limited transparency, insufficient case monitoring, inconsistent sanctions, weak coordination between campus units and external agencies, and institutional tendencies to prioritise reputation over victim-centred justice. The study contributes to policy discussions by offering a comparative account of campus-based prevention and response practices and by recommending stronger governance, clearer accountability, survivor-centred procedures, and integrated monitoring systems to ensure safer university environments.

## Highlights

- The study examines the preventing and responding to sexual violence strategy against women in Indonesian State Universities.
- Each university in Indonesia has its strategy for participating in preventing and responding to sexual violence through the PSGA and task force teams of PPKS, including making regulations on sexual violence and standard operating procedures, providing sexual violence education in the classroom, socialization, student gender organization, forming Focal Points for each Faculty, a story friend group organization, and increase religious activities such as *tahlil* and religious lectures.
- There are several processes that each university carries out in responding sexual violence, including reporting protection, assistance, prosecution, and victim recovery, such as medical recovery, psychological recovery, legal aid, and academic assistance.

The forms of participation and strategies adopted by universities in Indonesia demonstrate considerable variation in responding to cases of sexual violence. Such variation is evident in their preventive and remedial mechanisms, bureaucratic frameworks, and the quality of human resources. Establishing an effective and conducive bureaucratic system, supported by sufficient facilities, competent personnel, and qualified stakeholders, is essential to foster a safe campus environment and to realize the objectives of the Tridharma.

The implementation of higher education, as regulated in Act No. 12 of 2012 concerning the Tri Dharma of Higher Education, particularly Articles 6 and 9, emphasizes that higher education institutions are obliged to conduct teaching, research, and community service (Act No. 12 year 2012). Community service refers to initiatives designed to assist society in various aspects without financial compensation. Universities across Indonesia organize such programs to contribute to social welfare and national development (Direktorat Riset dan Pengabdian Masyarakat, 2020). One essential form of community service involves addressing the growing issue of sexual harassment and violence within educational environments and the broader community. Academic institutions are therefore expected to play an active role in promoting social welfare and ensuring a safe and supportive environment for all members of society (Arifin, 2010).

## The Phenomenon of Sexual Violence Cases

Sexual violence is not limited to physical sexual acts but also encompasses attempts to commit such acts through coercion, threats, abuse of power, or by displaying sexual materials, such as photos or videos, that are explicitly rejected by the victim (Afifah et al., 2019, p. 36). According to the 2020 Annual Report of the National Commission on Violence Against Women in Indonesia, a total of 299,911 cases were recorded. Among the 8,234 cases directly handled by the Commission, domestic violence and sexual violence were the most prevalent (Mustafainah et al., 2021).

In East Java Province, Indonesia, data from the Head of the Women's Empowerment, Child Protection, and Population Service indicated a significant increase in cases of violence against women, rising from 1,600 cases in 2020 to 1,887 cases in 2021. The majority of incidents involved sexual violence, accounting for 742 cases (39.32%), followed by physical violence with 618 cases (32.75%) and psychological violence with 532 cases (28.19%) (Kurnia, 2021). Furthermore, data from the Office for the Empowerment and Protection of Women, Children, and Family Planning of East Java Province revealed that Jember Regency recorded the highest number of cases in 2020, with a total of 2,010 cases. This high incidence is associated with a predominance of informal employment and the generally low quality of human resources, particularly in terms of educational attainment and household economic conditions (Hatta, 2020).

Acts of sexual violence not only occur in vulnerable places, but nowadays, it is more common in educational settings, which are supposed to be safe and just places to gain knowledge. There are many cases of sexual violence (Hoebel et al., 2022). The National Women's Commission noted that from 2012 to 2020, there were 45,069 cases of sexual violence in educational settings (Tardi, 2021). Throughout 2023, data from the National Commission on Violence Against Women indicated that sexual violence accounted for the highest number of cases, totaling 15,621, followed by psychological violence with 12,878 cases, physical violence with 11,099 cases, and other forms of violence with 6,807 cases. The analysis also revealed that individuals within educational institutions were the group most likely to experience violence (Elsa & Roren, 2024).

An effective strategy for responding sexual violence cases starts with prevention, not just dealing with victims, to reduce the occurrence of further victims. Law enforcement in preventing and dealing with sexual violence is important. However, Indonesia’s regulations on sexual violence had not been comprehensively integrated, such as in the Criminal Code, the Law on Child Protection, the Law on the Elimination of Domestic Violence, the Law on the Elimination of Criminal Acts, and Human Trafficking. Therefore, a special law is needed to regulate sexual violence rule starting from the process of prevention, responding, and legal sanctions.

Several published studies have some similarities with this study, but have differences in several aspects, including a study entitled *Efforts to Prevent Sexual Violence in the Educational Environment of Gorontalo State University*, this study focuses on testing the implementation of the Minister of Education Regulation Number 30 of 2021 concerning the Prevention of Sexual Violence at Gorontalo State University. The study entitled "*The New Oasis": Implementation of the Minister of Education and Culture Regulation on the Prevention and Handling of Sexual Violence in Higher Education*", this study focused on examining the extent of the implementation of the Minister of Education and Culture Regulation Number 30 of 2021 in higher education institutions in Indonesia with data analysis using desk studies by reviewing academic literature at each university but did not mention which universities were being the objects of the study (Simanjuntak & Isbah, 2022). The last research project focused on Prevention and Responding Strategies for sexual harassment and violence through peer support psychoeducation. This project, conducted at Halu Oleo University, studied strategies for preventing and responding sexual violence using peer support psychoeducation. A total of 15 students were tested in this study (Marhan et al., 2022). This study aims to fill the gap in previous studies related to sexual violence in higher education. Although there have been several previous studies that have examined sexual violence in higher education, none have specifically examined the specific strategies that have been carried out in preventing and responding sexual violence in several campuses in Indonesia using a case study approach. According to several published studies, there are differences in various aspects of the latest studies discussed. This study not only focuses on studying the prevention and responding strategies for sexual violence in one university, but several universities that have cases of sexual violence that are samples in this study. This study is an empirical study where the results of the study explain the phenomena that occurred in several universities.

Based on these problems, the author will examine sexual violence cases in several state universities in Indonesia and the strategies for preventing and responding these cases. This can be used as a reference for the government to make policies that provide space and encourage educational institutions to participate in sexual violence prevention activities, such as establishing regulations that contain strategies for prevention and responding in universities.

## The Role and Function of Higher Education

Law Number 12 of 2012 concerning Higher Education explained that the level of Higher education is secondary education, which includes diploma programs, undergraduate, master’s, doctoral, professional, and specialist programs carried out by higher education institutions based on the educational culture in Indonesia. Indonesia’s regulations on sexual violence had not been comprehensively integrated, such as developing the inner potential to deepen religious spirituality, self-control, personality, intelligence, noble character, and the skills needed by society.

Science uses a systematic approach based on scientific methodology to explain natural or social conditions in society. Technology in science is the use and utilization of various branches of knowledge that will produce value for society's needs and development.

Higher Education has the right to carry out learning and teaching, Research, and Community Service. Universities are free to develop science and technology to create a golden generation. Therefore, higher education institutions can freely develop a beneficial academic culture as a wise scientific group to organize interactions and associations in the international sphere to elevate the nation's dignity.

The functions of higher education institutions in Article 4 of Law Number 12 of 2012 include: a). To develop capabilities and shape the character and civilization of a dignified nation. b). To create an innovative, responsive, creative, skilled, competitive, and cooperative academic community through the Tridharma implementation c). To develop science and technology by applying humanities values (UU No. 12 Tahun 2012).

With these functions, institutions have the goal to realize community service and research to improve the nation’s welfare. Universities are expected to develop prosperous natural and human resources and to overcome the current problems happening in society, such as sexual violence. Law enforcement against sexual violence requires support from all aspects, including the community and universities, especially as educational institutions.

The rise of sexual violence cases has become a worrying paradox in universities. Educational institutions have duties and responsibilities to implement and transform knowledge for students. Academic institutions create a healthy, safe, and inclusive academic environment and provide exemplary, professional, responsible, and innovative educators and staff to produce quality, ethical, and moral students.

## Method

### Research Design and Setting

This study employed a descriptive qualitative case-study design to examine how Indonesian state universities prevent and respond to sexual violence against women, in which the author provides a comprehensive description of the data and focuses on an in-depth understanding of social phenomena and human behaviour within universities (Rustamana et al., 2024). All participants involved were selected using purposive maximum variation sampling (Nyimbili & Nyimbili, 2024). The researcher chose participants based on their relevance and involvement in the phenomenon under study. This sampling technique was guided by the researcher’s assessment of which characteristics were most pertinent to the research objectives. The research sites comprised state Islamic universities under the Ministry of Religious Affairs and state universities under the Ministry of Education, Culture, Research, and Technology. The institutions included the State Islamic University of Kiai Haji Achmad Siddiq Jember, the State Islamic University of Sunan Ampel Surabaya, the State Islamic University of Maulana Malik Ibrahim Malang, the State Islamic Institute of Kediri, the State Islamic University of Alauddin Makassar, the University of Jember, the State University of Surabaya, Airlangga University, Universitas Gadjah Mada, Brawijaya University, Sriwijaya University, the State University of Gorontalo, and the University of Trunojoyo Madura. These sites were selected because they represent different institutional models, regulatory frameworks, and campus-based responses to sexual-violence cases.

The unit of analysis was the university as an institution, with particular attention to the units responsible for prevention and case responding. In state Islamic universities, these functions are generally coordinated through Centres for Gender and Child Studies (PSGA) and integrated service units, while in other state universities they are primarily implemented through Task Forces for the Prevention and Response to Sexual Violence (PPKS). Participants were selected through purposive maximum-variation sampling to capture diverse perspectives from policy makers, service providers, lecturers, students, and campus actors involved in prevention, reporting, assistance, and recovery.

### Participants and Sampling

Participants included heads or representatives of PSGA, PPKS task-force members, legal-aid personnel, lecturers, students, and university units responsible for sexual-violence prevention and response. Inclusion was based on direct involvement in policy formulation, awareness programmes, reporting services, victim assistance, sanction procedures, or recovery support. Recruitment followed a purposive strategy and continued until the data obtained were sufficiently rich to describe the main institutional patterns. For journal submission, the manuscript should add the final number of participants, their institutional categories, gender composition where ethically appropriate, and the period of data collection.

### Data Collection Method

Data were collected through three complementary techniques: semi-structured interviews, non-participant observation, and document analysis. Semi-structured interviews were used to explore participants' views on prevention strategies, reporting procedures, institutional coordination, victim assistance, sanctions, and obstacles in implementation. The interview guide was prepared in advance but allowed follow-up questions so that participants could explain campus-specific practices in detail. Interviews were conducted face-to-face at the respective universities, lasted approximately 45 minutes, were audio-recorded with consent, and were transcribed for analysis.

Observation focused on the institutional environment, complaint mechanisms, awareness activities, and the practical arrangements used to prevent and handle cases. Document analysis covered rector regulations, circular letters, standard operating procedures, lecturer and student codes of ethics, activity reports, learning or socialisation materials, and relevant print and online news reports. Triangulating interviews, observations, and documents enabled the researchers to compare formal policy provisions with actual implementation across institutions.

## Results

### The Types of Sexual Violence in Indonesian State Universities

Sexual violence is gender-based violence that is not limited to sexual behavior or attempts to commit sexual acts that harm other people (Secretary of State for Foreign, Commonwealth & Development Affairs, 2022), such as women or girls by force, threats with violence, abuse of violence, and giving sex shows, whether photos or videos, which was strongly rejected by the victim (Afifah et al., 2019). The World Health Organization states that sexual violence is any sexual act, attempt to obtain a sexual act, unwanted sexual comments or advances, or acts to traffic, or otherwise directed, against a person’s sexuality using coercion” (Muzyamba, 2022).

The vast majority of those affected are women and girls (they are considered weak and unable to put up a fight), such as early childhood, school children, abandoned women, women with disabilities, minority groups (racial, religious, ethnic, and sexual), and other vulnerable groups. In the law, there is no clear, firm, and detailed definition regarding acts of sexual harassment or violence. For example, in the Criminal Code, this includes immoral acts, such as rape and obscenity.

The National Commission on Women divides the types of sexual violence into 15 forms (Ahsinin, 2015), which are: a. Rape, b. Sexual harassment, c. Sexual exploitation: This act relates to the women’s position to their marital status, that they have no bargaining value, apart from complying with the perpetrator's wish to be married (Graham Davies et al., 2018). This also applies to similar treatment of children, such as using them to fulfill sexual desires and forced marriages (Kementerian Sosial Republik Indonesia, 2020), d. Sexual abuse, e. Sexual slavery, f. Sexual intimidation, such as threats or attempted rape, g. Forced prostitution, h. Forced pregnancy, i. Forced abortion, j. Coercion of marriage, k. Women trafficking, l. Sexual control, such as forced clothing and criminalization of women through discriminatory rules based on morality and religion, m. Inhuman and sexual punishment, n. Traditional practices with sexual nuances that harm or discriminate against women, o. Forced contraception/sterilization.

In the regulation of the Minister of Religion no. 73 of 2022, 16 forms included sexual violence such as verbal, physical, and sexual violence in cyberspace. In Chapter 2, Article 5, Paragraph 1 states that the forms of sexual violence include acts committed verbally, non-physically, physically, or through information and communication technology, those are speech that insults the victim's body condition or gender identity, making advances, jokes, and whistling with sexual overtones, persuading, promising, offering something, threatening, or forcing the victim to engage in sexual activity, staring at other people with sexual overtones, peeking at other people who are doing private activities, deliberately showing genitals, touching, rubbing, holding, hugging, kissing, and rubbing his body on the victim's body, performing attempted rape, committing rape includes penetration with any part of the body other than the genitals, practicing a culture that contains sexual violence, forcing the victim to have an abortion, allowing sexual violence to occur, giving sexual punishment, sending sexual messages, jokes, pictures, photos, audio, or video to victims, taking, recording, uploading, and distributing photos and audio recordings of victims with sexual overtones, committing other acts of sexual violence under the provisions of the regulations.

Meanwhile, in the Minister of Education and Culture Regulation no. 55 of 2024, forms of sexual violence are explained in Article 5 that sexual violence includes actions carried out verbally, non-physically, physically, or through information and communication technology. Verbal and online actions are included in sexual violence. These actions are considered trivial even though they have a psychological impact on the victim and limit the right to education or academic work (Permendikbudristek Number 55 of 2024). Furthermore, Article 5, Paragraph 2, explained in detail why the sexual violence forms are not different from the sexual violence forms in the regulation of the Minister of Religion number 73 of 2022.

The regulation comprehensively outlines actions that constitute sexual harassment, including making discriminatory remarks about an individual’s physical appearance or bodily condition; engaging in physical contact such as touching, groping, or rubbing that causes discomfort; whistling; and sending text messages, photos, images, or videos of a sexual nature. Such behaviors are classified as acts of sexual harassment and are subject to sanctions or legal punishment for the perpetrators. Furthermore, individuals who knowingly permit or fail to report incidents of sexual violence are also deemed to have participated in such acts and may face similar penalties. Cases of sexual violence in Indonesian universities manifest in various forms and levels of severity, ranging from minor to serious offenses.

### Factors Causing Sexual Violence in Higher Education

Many types of sexual violence may occur on campus, including verbal, physical, and non-physical harassment. Verbal harassment includes harsh or demeaning words, pressure to engage in sexual activity, and unwanted sexual comments. Physical harassment includes touching, kissing, or more serious acts such as rape, while non-physical violence includes sending sexually suggestive texts, photos, or videos, or seducing through online chat applications. The following is a description of the triggers for sexual violence on several campuses, including:

- *Patriarchal culture remains deeply ingrained in society*: This system of thought positions men as the primary holders of power and dominance across all sectors of life, including the family, economy, politics, law, and education. Such a culture renders women vulnerable and easily exploited. Patriarchy not only deprives women of their rights but also confines them within an unequal social structure.
- *Social norms justify acts of sexual violence*: In some communities, sexual harassment is seen as a common occurrence. Victims are frequently blamed and stigmatized by society. Within educational institutions, such cases are often hidden to protect the institution’s reputation, forcing victims to endure the injustice.
- *Ineffective education about sexuality*: The Indonesian curriculum does not include comprehensive sex education as a mandatory subject. Discussions about sexuality are still considered taboo and inappropriate (Supriansyah, 2023). Consequently, children lack a proper understanding of how to meet their biological needs healthily and lawfully.
- *The power gap between the perpetrator and the victim*: Sexual violence that occurs in several universities is often driven by an imbalance of power between the two parties. The authority associated with a higher position enables the perpetrator to dominate the victim through various threats (Saputra et al., 2024). In many cases, information about such incidents in higher education institutions is concealed to protect the university’s reputation.
- *The campus environment and facilities with poor lighting and supervision*: Some incidents of sexual violence or harassment in higher education occur in vulnerable locations that are not visible to others. Such as corners of buildings, bathrooms, closed spaces, and gardens.

### The Strategy of Sexual Violence Prevention in Indonesian Universities

The emergence of violence against women is intricately connected to cultural ideologies and social values embedded within societal structures and gender relations. In the contemporary era, numerous crimes, particularly those targeting women and involving sexual violence, have become increasingly prevalent. Technological advancement is considered one of the contributing factors to these incidents, with minors and adolescents often identified as the primary victims. Such violence occurs across diverse social settings, from traditional communities to urban environments that embody modernity. Within educational institutions, numerous cases of sexual violence have been documented, perpetrated by lecturers against students, students against lecturers, and among students themselves. This phenomenon has attracted significant attention from both mass and social media, reflecting its growing visibility and urgency as a social issue (Sumintak & Idi, 2022, p. 57).

Using the law as an instrument for preventing sexual violence in universities is a rational and necessary effort. It serves both as a foundation and as a guarantee of legal certainty for the actions taken. As a form of deviant social behaviour, acts of sexual violence can be addressed through various approaches, including law, religion, morality, customs, education, the arts, the media, and exemplary leadership. The application of legal instruments becomes even more strategic when linked to the high prevalence of sexual violence. Such instruments can offer protection and legal certainty if they include strict sanctions for offenders (Hartono, 2022).

Various problems of sexual harassment that occur in higher education can be reduced in several ways. Universities must be able to recognize the reality that there is sexual harassment behavior happening, which can then worsen academic potential and mental health. By dealing with the problem of sexual harassment on campus through intervention and prevention, universities can indirectly provide messages to the academic community regarding appropriate gender, racial, and sexual norms (Ishak, 2020). Based on reports from the National Academies of Sciences, Engineering, and Medicine (NAS) regarding sexual harassment behavior that occurs in educational settings. In its report, the NAS provides four recommendations that can end sexual harassment, namely integrating the diversity and inclusion values into policies and procedures; changing power dynamics to relieve reliance on advisory relationships; providing support to survivors of sexual harassment through services and reporting that will minimize the risk of retaliation by the perpetrator; and making improvements in accountability and transparency (Ishak, 2020). In addition, it appears that the obstacle to prevention, response, and recovery for victims of sexual violence is impunity for perpetrators in the educational environment itself, which provides more protection for perpetrators to maintain the good name of the institution (Chusna Farisa, 2020).

To find out and establish effective strategic policies, it is necessary to know the root causes of sexual violence on campus. Several factors lead to sexual violence on campus, including there are opportunities to commit acts of sexual violence, such as boarding houses against residents. Campus lighting facilities at night were inadequate, causing the perpetrators to take advantage of the situation, Sexual violence by colleagues with smartphone features such as video calls, Power players in the sense that sexual violence is carried out with the lure of certain benefits to victims, conducted by the lecturer by giving questions when the exam is not in the context of the subject being studied, which leads to a certain sensitivity.

Preventive efforts carried out by various tertiary institutions are more massive in the socialization of sexual violence. In addition, at the prevention and responding stage, it is necessary for tertiary institutions to strive for counselors for sex education counseling purposes through several specific steps so that the level of effectiveness is quite high to reduce cases or even the absence of sexual violence cases in tertiary institutions, namely:

- *Analysis*, in this stage, the counselor must formulate a formulation of the problem and collect data about sexual violence. In this stage, the problem will be known, and the hypothesis must be determined quickly.
- *Synthesis* is the process of summarizing or sorting the data from the analysis process on the sexual violence issue. The summarized data should make it easier to understand and not complicate the process. This step is important because it is the first step in the counseling process itself.
- *Diagnosi*s is the main step. In this step, the discussion will focus more on the issue of sexual violence, its causes and effects, and the results of the analysis. In this step, the counselor will get a method for the counseling process. The choice of counseling method is important, considering that the psychological background of each person is different.
- *Counseling* is a process where the counselor must convey input, solutions, or directions obtained through the process. This step is a real action in the form of socialization. Counseling could be successful if there is no conflict in a thought. At this stage, clients who are victims of sexual violence are expected to make decisions to develop and maximize their potential.
- *A follow-up* is an optional intervention that may be conducted by the counselor when the client demonstrates limited understanding of the problem or has not yet succeeded in resolving it (Wulandari & Suteja, 2019).

The prevention of sexual violence against women and children that can be carried out by universities includes the following activities: Communication, information, and education about the prevention and responding of sexual violence; The formulation of sexual violence prevention policies and training on the prevention and responding of sexual violence. Meanwhile, the government's strategy to reduce sexual violence against children can be done by making legislation and implementing policies that protect children from all forms of sexual violence, which are more concrete in terms of text and context.

Strategic policies for prevention and response should be developed based on campus-specific data. The point of emphasis is on the context of how a policy is analyzed by academics (academic policy analysis) and studied by practitioners (applied policy analysis). Thus, strategic policies on some of the statements above can be effective policies in implementation in tertiary institutions.

In the policy context taken by tertiary institutions to ensure the internal security of the campus from sexual violence against women and children, it can be programmed from various aspects, namely:

- *Health sector*: increasing the capacity of health workers related to sexual violence so that health workers can provide services based on the principle of fulfilling the rights of women victims of sexual violence.
- *The social sector*: ensures the availability and prepares counselors with a human rights and gender perspective to assist women victims of sexual violence, including rape victims, including assistance during pregnancy as long as the rape victim is willing to continue her pregnancy.
- *The religious sector:* building the capacity of religious leaders and counselors so that they have a human rights and gender perspective in assisting women and children victims of sexual violence.
- *The legal sector*: provides protection and immediate legal treatment of women and children victims of sexual violence.

Indonesia has approximately 2,694 private and public tertiary institutions spread throughout the country. Of the thousands of universities, not all of them have clear rules regarding the responding to cases of sexual violence. This can be a fertile field for the emergence of various cases of sexual violence because the perpetrators feel that there are no rules that can penalize them. It is possible that so far there have been many victims of sexual violence that have occurred on campus but cannot be prosecuted, and only end in peace. Policymakers on campus should act immediately with the passing of the Minister of Education and Culture on Sexual Violence on Campus so that student safety is guaranteed and the teaching and learning process does not go wrong (Simanjuntak & Isbah, 2022).

In the replacement of Permendikbudristek No. 30 of 2021 with Permendikbudristek No. 55 of 2024 regulate how higher education is responsible for preventing sexual violence. Higher education is one of the parties that has a big role because higher education is a formal institution to provides academic learning that involves soft skills. Some Efforts must be made by tertiary institutions to make policies on sexual harassment, make complaint submission procedures, formulate codes of ethics, provide materials in orientation programs, conduct workshops, and provide pocketbooks (Saraswati & Sewu, 2022).

Sexual violence prevention strategies have been implemented by several universities in Indonesia. These strategies demonstrate the commitment of higher education institutions to creating a safe, inclusive, and sexual violence-free campus environment. The efforts include establishing Task Forces for the Prevention and Responding of Sexual Violence, issuing rector’s circulars or regulations, preparing standard operating procedures, providing complaint and legal aid services, and conducting outreach and education for students and lecturers. In addition, several universities have also developed integrated security systems, learning modules, sexual violence surveys, and peer-support groups as part of more sustainable prevention measures. The strategies implemented by each university are summarized in Table 1.

**Table 1:** The Sexual Violence Prevention Strategies Implemented by Each University

University	Sexual Violence Prevention Strategies
**University of Brawijaya**	There is the rector's statement letter number 70 of 2020 There are consulting and legal aid institutions There is a Task Force for the Prevention and Responding of Sexual Violence Socialize about preventing and responding sexual violence
** University of Gadjah Mada**	There is a Task Force for the Prevention and Responding of Sexual Violence Socialize about preventing and responding sexual violence There is a Standard Operating Procedure for preventing and responding sexual violence Build a clean and safe environment Build an integrated security system
**University of Airlangga**	There is Airlangga Inclusive Learning (AIL) There is a Task Force for the Prevention and Responding of Sexual Violence There is a Help Center service
**State University of Surabaya**	There is a Task Force for the Prevention and Responding of Sexual Violence Create a guidebook related to preventing sexual violence Socialize about the dangers of sexual violence
**State University of Jember**	There is the rector's statement letter number 04 of 2022 There is a Task Force for the Prevention and Responding of Sexual Violence Take a healthy walk by calling for the rejection of sexual violence Disseminate information to students and lecturers about how to prevent and handle sexual violence
**The State Islamic University of Maulana Malik Ibrahim**	There is the rector's statement letter number 4748/Un 3/HK.00.5/08/2018 regarding the student code of ethics Wrote guidelines for preventing and responding sexual violence in collaboration with a student crisis center Standard Operating Procedure of the Prevention and Responding of Sexual Violence Establishment of integrated unit services There is a story friend group organization
**The State Islamic University of Sunan Ampel**	There is a Task Force for the Prevention and Responding of Sexual Violence Socialize the Ministry of Religion regulations regarding the prevention and responding of sexual violence Establish a vocal point group for each faculty
**The State Islamic University of Kiai Haji Achmad Shiddiq**	There is the rector's statement letter number 315 of 2020 about Prevention and Responding of Sexual Violence Standard Operating Procedure of Prevention and Responding of Sexual Violence Socialize about preventing sexual violence
**The State Islamic Institute of Kediri**	There is the rector's statement letter number 532 of 2020 about Prevention and Responding of Sexual Violence Establish an integrated service agency Establish a vocal point group consisting of lecturers and students Standard Operating Procedure of Prevention and Responding of Sexual Violence
**University of Sriwijaya**	There is a Task Force for the Prevention and Responding of Sexual Violence Socialize about preventing and responding sexual violence, such as the seminar Hello Sister held by the South Sumatra American Alumni Community PPKS module in e-learning Conducting a sexual violence survey
**University of Trunojoyo**	Ratify the chancellor's regulations on PPKS Providing sexual violence reporting services Forming a PPKS task force team Forming an SAHABAT Task Force team Socializing reproductive health education, sexually transmitted diseases, and teaching the limits of sexual activity during a child's development Periodically disseminate information regarding guidelines for preventing and responding sexual violence
**State Islamic University of Alauddin Makasar**	Socializing the Integrated Service Unit for Prevention and Responding of Sexual Violence (ULT PPKS) Providing education about violence prevention Creating a healthy, comfortable, and safe campus environment There is the rector's statement letter

In Permendikbud No. 55 of 2024 concerning PPKS, Article 6 also explained prevention strategies that must be carried out by universities. 3 aspects must be carried out in preventive action, through learning, strengthening governance, and strengthening the culture of the student community, educators, and education staff.

Universities in Indonesia generally have prevented sexual violence acts either through strengthening governance, such as compiling rules and standard operating procedures for preventing sexual violence, through outreach activities to students, lecturers, and staff through seminars, and in learning and curriculum development activities. Not only that, each tertiary institution has a different mechanism for preventing sexual violence according to the types of cases that often occur and the available infrastructure. In Chapter III, Article 6, Paragraph 1, PMA no. 73 of 2022 concerning PPKS explained that educational institutions are required to prevent sexual violence through outreach, learning, strengthening governance, strengthening culture, and other activities according to needs.

Prevention through outreach activities is carried out by conveying information, campaigns, and other forms related to sexual violence. Prevention through learning activities is carried out through curriculum development and learning; creation of modules, books, and other literature; and organizing training, halakah, studies, and other activities. Prevention through governance strengthening activities includes standard operational procedures preparation for the Prevention of Sexual Violence; provision of facilities and infrastructure according to needs; and cooperation with related agencies. Prevention through cultural strengthening activities is carried out through environmental awareness, care for the prevention of sexual violence, and the development of communication networks. In implementing the prevention of sexual violence, educational institutions can cooperate and coordinate with other institutions such as ministries, local governments, public or private universities, communities, and parents/guardians of students.

### Strategies and Mechanisms for Responding Sexual Violence in Higher Education

The law explained the mechanism for responding to sexual violence cases. it stages include reporting, protection, assistance, prosecution (sanction), and recovery of victims in Permendikbud No. 55 of 2024 the replacement of Permendikbudristek No. 30 of 2021. However, apart from implementing these regulations, each university has its own way of responding these cases. Such as assisting victims' psychological recovery. Because such cases may harm victims’ mental health and future prospect. Such as the State Islamic University of Sunan Ampel Surabaya, the State Islamic University of Maulana Malik Ibrahim Malang, the State Islamic University of Jember, and the State Islamic Institute of Kediri forming a focal point consisting of students and lecturers to accompany and provide direction in listening to victims' complaints, and the team is committed to maintaining their good name and helping to resolve the problem. At Airlangga University, there is Inclusive Learning, which helps provide justice for students and lecturers affected by cases. At the University of Surabaya, Brawijaya University, and State University of Jember, in recovering the victims by providing personal assistance, such as providing religious guidance, motivation for the future through consulting institutions, and legal assistance in collaboration with psychologists.

In responding to sexual violence, tertiary institutions are required to carry out the Responding of Sexual Violence. The stages of responding to sexual violence include reporting, protection, assistance, prosecution, and recovery of victims. This has been regulated in PMA No. 72 of 2022 concerning PPKS for educational units under the auspices of the Ministry of Religion. Meanwhile, in Permendikbud No. 55 of 2024 concerning PPKS for state education units under the auspices of the Ministry of Research, Technology, and Higher Education, the responding stages include assistance, protection, imposition of sanctions, and recovery of victims (Permendikbudriset No. 55 Tahun 2024). The following are the stages in dealing with sexual violence, including:

- *Reporting*: The report must contain information on the identity of the Reporting Party, the identity of the Victim, the identity of the alleged perpetrator, the type of sexual violence that occurred, and the time and place of the incident. Assistance in this case is given to victims or witnesses who have status as students, educators, educational staff, and campus residents in the form of a. counseling; b. health services; c. legal assistance; d. advocacy; and/or e. social and spiritual guidance. In terms of implementation of assistance must obtain the consent of the victim.
- *Protection*: In terms of protection for victims or witnesses, each university provides the following things; a) Guarantee of continuity to complete education for students; b) Guarantee of continuity of employment as educators and education personnel at the higher education concerned; b) Guaranteed protection from physical and non-physical threats from perpetrators or other parties or the repetition of sexual violence in the form of facilitating reporting of physical and non-physical threats to law enforcement officials; c) Protection of identity confidentiality; d) Provision of information regarding protection rights and facilities; e) Provision of access to information on the implementation of protection; f) Protection from the attitudes and behavior of law enforcement officers that reduce and strengthen the stigma against victims; g) Protection of victims and reporters from criminal charges; h) Civil lawsuits over reported incidents of sexual violence; i) Provision of safe housing; j) Protection of security and freedom from threats related to the testimony given.
- *Persecution and Sanctions*: Several types of sanctions can be imposed on perpetrators of sexual violence. It is explained the types of administrative sanctions including: minor administrative sanctions; moderate administrative sanctions; and severe administrative sanctions.
- Table 2 presents the forms and types of administrative sanctions that may be imposed on parties who commit violations within the higher education environment. These administrative sanctions are classified into three categories: minor, moderate, and severe administrative sanctions. Each category has different forms of sanctions according to the level of violation committed. This classification aims to provide clarity regarding the administrative consequences that may be applied proportionally, fairly, and in accordance with the applicable laws and regulations.

**Table 2:** Forms and Types of Administrative Sanctions

Types of Administrative Sanctions	The Forms of Sanctions
**Minor administrative sanctions**	Written warning A written statement of apology that is published internally on campus or in the mass media
**Moderate administrative sanctions**	Temporary dismissal from office without obtaining office rights; Reduction of rights as a student includes: Postponement of lectures (suspension); Scholarship revocation; Deduction of other rights
**Severe administrative sanctions**	Permanent dismissal as a student Permanent dismissal from the position as Educator of Education Personnel, or Campus Residents, under the provisions of laws and regulations, from the Higher Education concerned

In this stage, the victim is provided with assistance in legal recovery. The legal process is as follows:

**Figure:**
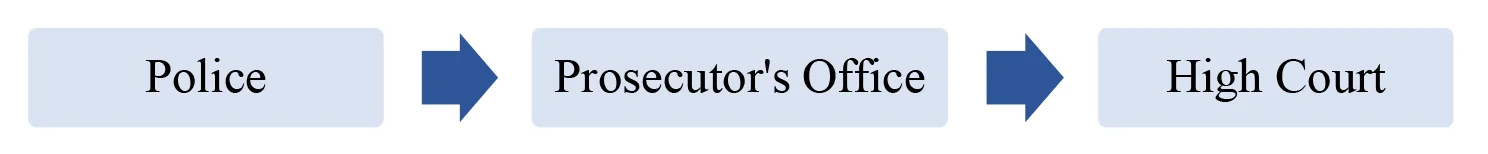


*Victims' Recovery:* The recovery of victims in the form of medical treatment, physical therapy, psychological therapy, and social and spiritual guidance. In the recovery process, the education unit can coordinate or involve competent special personnel, such as a. doctors or other health workers; b. counselor; c. psychologist; d. public figure; e. religious leader; f. other companions as needed, including the needs of Victims with disabilities (Permendikbudristek No. 55 Tahun 2024).

Every University is required to have a task force for responding sexual violence. Membership of the Task Force comes from the relevant tertiary institution, consisting of educators, educational staff, and students. There are several aspects in the victim recovery process, including:

- *Medical assistance and recovery*: this medical recovery is provided to victims who need physical examinations, medical procedures, and treatment. Apart from that, it is provided when the victim wants to undergo a physical examination or a post-mortem to handle the case.
- *Psychological assistance and recovery:* this service is provided when victims need psychosocial intervention by psychologists and psychiatrists to strengthen the support system. This service is also provided if the victim wishes to undergo post-mortem psychiatry for case-responding purposes.
- *Legal assistance and recovery*: This assistance is provided to survivors who wish to resolve their cases through legal processes. This service aims to prepare survivors to seek justice through the legal process.
- *Academic assistance and recovery*: survivors are also given academic assistance to help them complete their studies. Academic assistance is provided by academic supervisors, supervisors, and other lecturers assigned to accompany them. This assistance includes assisting in completing pending assignments and assisting in carrying out lectures safely.

## Discussion

The findings from the data collected show that state universities in Indonesia have generally implemented prevention efforts as stated in the Minister of Education and Culture Regulation Number 55 of 2024 and the Minister of Religion Regulation Number 73 of 2022. There are several strategic steps to prevent sexual violence that have been carried out by several state universities in Indonesia, including:

### Strengthening Regulations

Regulations regarding the prevention and responding of sexual violence are fundamental instruments that must be made and ratified to minimize cases of violence in tertiary institutions. Regulations should be used as guidelines for tertiary institutions to formulate policies and take action to prevent and deal with sexual violence. The Minister of Education and Culture and the Ministry of Religion have issued regulations on the prevention and management of sexual violence specifically in the educational environment under them. The regulations are general and need to be translated into more technical regulations so that they are easily implemented by each agency. Every tertiary institution must have special regulations regarding the code of ethics for preventing and responding sexual violence, even in each study programmes and departments must also have technical rules so that they are easy to implement.

Almost all public universities in Indonesia have a task force for the prevention and responding of sexual violence, especially after the issuance of Minister of Education and Culture Regulation No. 55 of 2024 concerning the prevention and responding of sexual violence. The task force formed has to prevent acts of violence up to the responding stage if a case occurs in a tertiary institution. Meanwhile, Islamic State Universities also published Minister of Religion Regulation Number 73 of 2022. With this regulation, every chancellor at the university issues a chancellor's regulation that applies to each University.

### Socialization

Prevention of sexual violence in tertiary institutions through socialization and booklets is an important step in providing an understanding of sexual violence forms in tertiary institutions contained in the regulations of the Minister of Education and the Ministry of Religion. The socialization was carried out online or offline as well as through visual and printed information media with various activities and programs for students, students and educators within the Universities.

Each tertiary institution has a different way of carrying out this socialization. The socialization program at the State Islamic University of Malang through an exhibition of forms of action that fall under sexual violence and the types of sanctions. At the University of Jember, one of the socialization programs is through casual walks by some students while saying or shouting "stop sexual violence" along the way. Majority of state universities such as the State Islamic Institute of Kediri, Airlangga University, The State University of Surabaya, The State Islamic University of Alaudin Makassar, Sriwijaya University, Gadjah Mada University, Hasanuddin University, the State Islamic University of Syarif Hidayatullah, University of Indonesia, State University of Gorontalo, Sriwijaya University and University of Trunojoyo conduct outreach through workshops, seminars and special publications about women and children. Apart from that, online socialization on the prevention of sexual violence has been carried out by the majority of universities in Indonesia, such as through social media Instagram, Facebook, Telegram, and Twitter.

### Gender Responsive Education Curriculum

The curriculum is a system of plans and arrangements regarding teaching materials that guide the learning and teaching process. The curriculum is described in components consisting of learning objectives, materials and lecture topics, teaching materials or references used, learning strategies, media or facilities and infrastructure used, and evaluation. Educational institutions that pay attention to gender equality will include these gender equality efforts as part of their vision and mission, which will then be implemented through the curriculum (Tafsir, 2006). Several universities in Indonesia have implemented a gender-responsive education curriculum in which gender issues are included explicitly in every teaching material. These teaching materials were then developed with gender-responsive materials and implemented for students to practice in everyday life.

Implementing a gender-responsive curriculum in tertiary institutions uses a contributions approach where issues concerning gender are included explicitly in the existing curriculum. This model approach has been applied, among others, at The State Islamic University of Maulana Malik Ibrahim Malang, The State Islamic University of Kiai Haji Achmad Siddiq Jember, The State Islamic University of Sunan Kalijaga Yogyakarta, The State Islamic University of Syarif Hidayatullah, and the University of Indonesia. At the State Islamic University of Sunan Kalijaga Yogyakarta and The State Islamic University of Syarif Hidayatullah, the formulation of a curriculum with a gender perspective is reflected in various courses that present gender material. The results of formulating a curriculum with a gender perspective are a good achievement from the leadership's policy in the framework of building gender equality.

### Strengthening Governance

Strengthening institutional governance is an important factor in preventing and dealing with sexual violence. After the enactment of regulations on the prevention and responding of sexual violence in state higher education institutions in Indonesia, they have started to improve institutional governance, including: a) To formulate policies, all state tertiary institutions in Indonesia already have specific regulations regarding procedures and technical implementation of prevention and control of sexual violence within higher education institutions. b) Has specific guidelines on techniques for preventing and dealing with sexual violence. c) Establishing a special team in perpetrating and dealing with sexual violence in tertiary institutions. Each state university under the Ministry of Education and Culture has a special team or PPKS task force, while in state Islamic universities each university has a gender and child study center that handles all matters related to women and children including the prevention and responding of sexual violence. d) Have an Integrated Service Unit as a means of reporting cases of sexual violence that occur both online and offline. e) Cooperating with other agencies to prevent and deal with sexual violence. Each tertiary institution has collaborated with legal institution offices, such as the police and advocates in law enforcement. In the field of health, higher education collaborates with higher education health clinics, health centers, and the nearest hospital. Meanwhile, in psychology, universities cooperate with higher education counseling institutions and social services for women's empowerment and child protection. At the State Islamic University of Maulana Malik Ibrahim Malang, there is an institution called "Friends of Stories" in the counseling aspect. The University of Brawijaya carries out non-litigation assistance, there is a mental health test program for all academics to detect mental illness at Airlangga University.

### Strengthening Culture

Maintaining fair and civilized human values in the higher education environment is the same as maintaining the diversity of Indonesian culture in a single state included in Pancasila. The values of Pancasila are extracted from the inner feelings and intentions of people's lives in Indonesia. These cultural values and practices must also be applied in the higher education environment including a culture of respecting individual integrity, maintaining the dignity and worth of each individual such as not committing sexual violence in all its forms, positioning women and men in equal relations, respecting the integrity of each individual, both physically and emotionally by protecting and nurturing those who are younger and respecting those who are more mature. If the values contained in Pancasila are applied in everyday life, they will build and maintain culture for Indonesian citizens with customary life, divine, and human values.

The surrounding culture and environment must actively support and create space for victims of sexual violence. They should be encouraged to enhance their skills and provided with equal opportunities in both education and employment sectors. Establishing a safe and inclusive environment is essential for fostering personal development and enabling them to pursue and realise their future aspirations (Ogden & Tarpey, 2024).

### Strengthening Spirituality

In the spiritual strengthening stage, every tertiary institution in Indonesia has several activity programs by each faculty and student organization. State Islamic universities have integrated social sciences and religious sciences into each subject. For example, the Centre for Gender and Child Studies of the State Islamic University of Malang conducts religious studies in collaboration with student organizations. The PSGA of the Islamic Institute of Kediri holds a webinar on women and religion every week. In several campuses in other Islamic countries, they also often carry out religious deepening activities through the habit of reading prayers together before starting the learning and teaching process in class, joint *tahlil* activities, tolerance, and deepening character and morality under their respective religious beliefs.

There are two methods commonly applied by every tertiary institution to deepen religion, namely the method of habituation (*tazkiyat al-nafs*), and the method of practice or behavior coaching. This method has been applied in all tertiary institutions in Indonesia through classroom learning and the habit of being polite and courteous both when interacting and speaking directly or through social media with lecturers, fellow students, and all university employees as well as 3S habituation (smiling, greeting, and offering salutations). The 3S habituation is intended to teach students about the importance of maintaining friendly relations and establishing kinship as a family with lecturers, employees, and friends through smiling, greeting each other, and saying greetings to pray for kindness to others, whose value is also equivalent to worship.

### Character Strengthening

Methods that are carried out through socialization and certain booklets are fundamental efforts in the prevention process. Every university in Indonesia has a lot in common in terms of prevention and treatment. However, there are differences in the program carried out by the State Islamic Institute of Kediri in the arrangement of student activities; there is a special Gender UKM institution. In addition, the State Islamic University of Sunan Ampel Surabaya also has differences in the scope of implementation of the PSGA program and the PPKS Task Force, namely the existence of Focal Points placed at each Faculty in the hope of narrowing the space for potential sexual violence. At the State University of Surabaya, there is a spiritual deepening program and character development from an early age.

Meanwhile, in the process of responding sexual violence, every university has the same stages in cases of sexual violence. The steps that must be taken include reporting, protection, assistance, prosecution, and victim recovery. In these steps, the victims will be accompanied by the PPKS Task Force team or special teams at each university.

**Figure 1:**
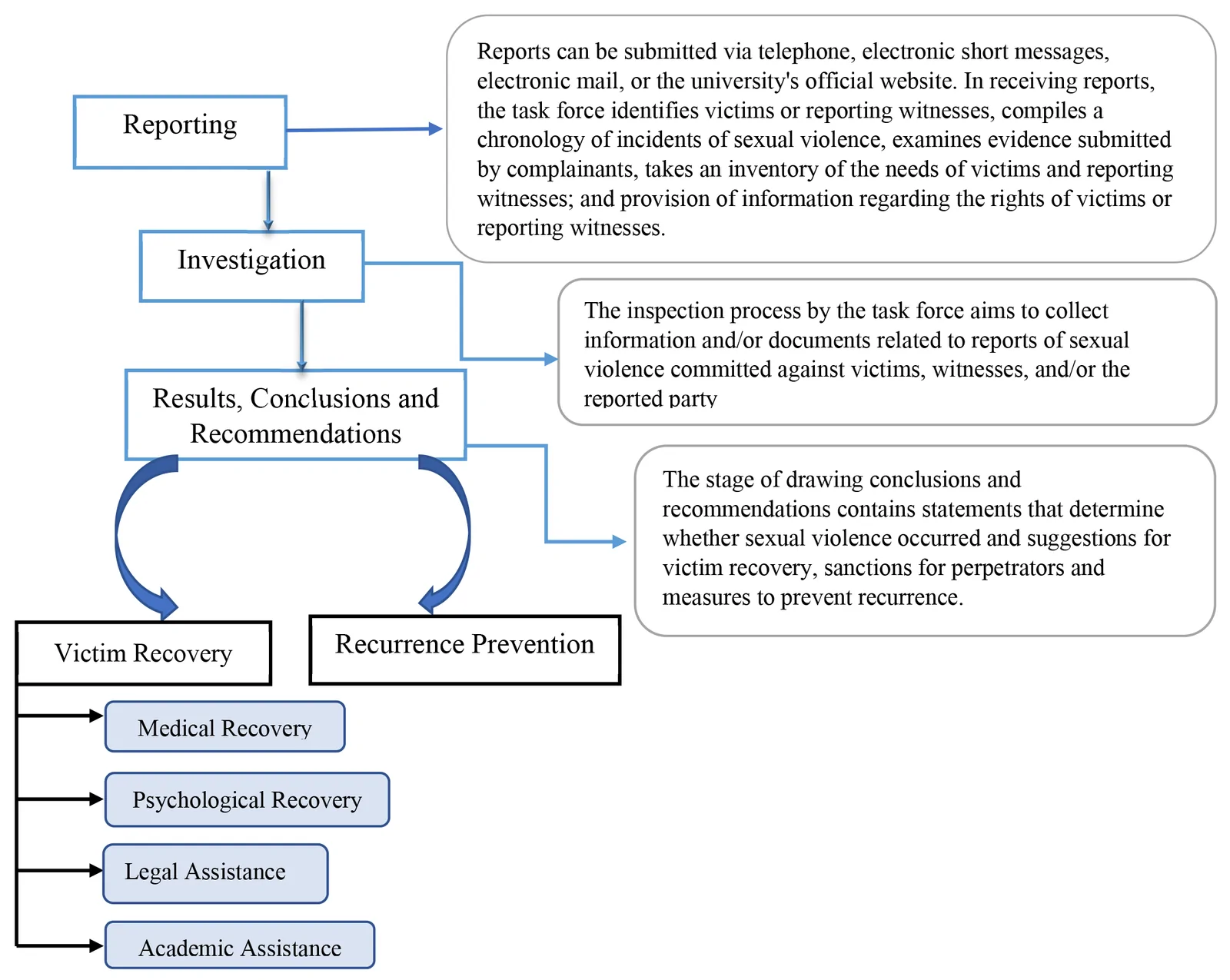
The Process and Stages of Responding to Sexual Violence

The process and stages of responding sexual violence that have been carried out by each university are summarized in Figure 1.

The regulations of the Minister of Education and the Minister of Religion on the prevention and responding of sexual violence are still weak. The regulations only explain technical articles on prevention and responding solutions; there is no detailed discussion of the sanctions that will be imposed by the perpetrators. Law number 12 of 2022 concerning criminal acts of sexual violence is also not discussed in detail, only discussing the process of investigation, prosecution, and examination in court in articles 52-64.

Sanctions for perpetrators of sexual violence are only found in the Criminal Code (KUHPidana); the regulation does not use the term sexual harassment/violence but uses the term indecency. Indecency is found in the second book on crimes, Chapter XIV on Crimes Against Morality (Articles 281 to 303). The article explains that the punishment for perpetrators of indecency is subject to a maximum sentence of 5 years in prison, and pays compensation for the losses experienced by the victim.

### Conclusion

The research highlights that sexual violence has recently become more frequent, and technological development has contributed to the increasing number of sexual-violence incidents that have occurred in Indonesian Universities. At least several universities have become places for perpetrators of sexual violence to carry out their actions, both students and lecturers. The most rational strategy for reducing the high number of cases of sexual violence is through regulations. This has been done through the Minister of Research, Technology, and Higher Education, and the Minister of Religion, to give legal certainty. apart from that, it is also assisted by the PSGA institution and task force teams of PPKS, including making regulations on sexual violence and standard operating procedures, providing sexual violence education in the classroom, socialization, student gender organization, forming Focal Points for each Faculty, a story friend group organization, and increasing religious activities such as *tahlil* and religious lectures.

There are several processes that each university carries out in responding sexual violence, including reporting, protection, assistance, prosecution, and victim recovery, such as medical recovery, psychological recovery, legal aid, and academic assistance. However, another problem that becomes the majority is in the implementation structure, which still has weaknesses, especially in the field of escorting cases, and is limited to administrative sanctions against perpetrators, and tends to be covered up to maintain university quality.

## Data Availability

The primary data that supports the findings of this study are available from the corresponding author upon reasonable request.
